# Mathematical modeling of self-contained CRISPR gene drive reversal systems

**DOI:** 10.1038/s41598-019-54805-8

**Published:** 2019-12-27

**Authors:** Matthew G. Heffel, Gregory C. Finnigan

**Affiliations:** 1 Division of Biology, 116 Ackert Hall, Kansas State University, Manhattan, KS 66506 USA; 2 Department of Biochemistry and Molecular Biophysics, 141 Chalmers Hall, Kansas State University, Manhattan, KS 66506 USA

**Keywords:** Evolutionary biology, Population genetics

## Abstract

There is a critical need for further research into methods to control biological populations. Numerous challenges to agriculture, ecological systems, and human health could be mitigated by the targeted reduction and management of key species (e.g. pests, parasites, and vectors for pathogens). The discovery and adaptation of the CRISPR/Cas editing platform co-opted from bacteria has provided a mechanism for a means to alter an entire population. A CRISPR-based gene drive system can allow for the forced propagation of a genetic element that bypasses Mendelian inheritance which can be used to bias sex determination, install exogenous information, or remove endogenous DNA within an entire species. Laboratory studies have demonstrated the potency by which gene drives can operate within insects and other organisms. However, continued research and eventual application face serious opposition regarding issues of policy, biosafety, effectiveness, and reversal. Previous mathematical work has suggested the use of modified gene drive designs that are limited in spread such as daisy chain or underdominance drives. However, no system has yet been proposed that allows for an inducible reversal mechanism without requiring the introduction of additional individuals. Here, we study gene drive effectiveness, fitness, and inducible drive systems that could respond to external stimuli expanding from a previous frequency-based population model. We find that programmed modification during gene drive propagation could serve as a potent safeguard to either slow or completely reverse drive systems and allow for a return to the original wild-type population.

## Introduction

The discovery of the CRISPR/Cas system as a powerful genetic editing biotechnology has revolutionized many fields across agriculture and biomedical research. To date, control of populations remains essential to managing of our food supply as well as preserving natural habitats and their diverse ecological composition. For instance, invasive species cause severe damage on a wide scale in many ecosystems^1–4^ and insects serve as vectors for transmitting an increasing number of diseases including Zika, dengue, malaria, Lyme disease, and typhus^5–7^. Current mechanisms to mitigate biological populations rely on a variety of strategies ranging from mosquito netting^8^ to natural predators^9^. However, no previous methodology has existed that could effectively alter an entire population on a global scale.

The demonstration that a nuclease-based “gene drive” (GD) could artificially propagate genetic information through a population could have profound impacts for human health and the environment. This mechanism utilizes a strategy within a single diploid cell to transform the heterozygous condition (between the pairing of a genetically modified organism (GMO) and a wild-type (WT) individual) to the homozygous condition resulting in *Super*-Mendelian inheritance of the desired genetic cargo and a rapid sweep through a population^10^ (Fig. S1).

The potential benefits and applications of such a system are numerous. The eradication of insect populations that serve as disease vectors could have profound impacts globally^11^. Successful gene drive systems have now been demonstrated within fungi, mammals, and insects^12–15^. The intended trait to drive through the species of choice is often to interfere with sex determination to bias the male-female ratio to an extreme to cause populations to crash^16–18^. However, challenges remain in the design and effective propagation of the drive within sample populations and ethical and ecological concerns regarding actual use of this system within wild populations remain.

Methodologies with traditional drive systems allow only two possible outcomes: (i) the GD runs to completion and takes over the population or (ii) the GD is removed from the population (via evolved resistance, sub-optimal fitness, or is destroyed, etc.). Therefore, we envisioned a GD system that could be tuned or modified while it was actively spreading and already present within a population. The rationale for this type of programmable system includes issues of biosafety, tunability, and customization. In terms of design, any added components would be installed within the GD itself, proximal to the nuclease and guide RNA(s) or at additional loci. Previous studies have highlighted numerous ways that the activity of nucleases could be controlled and have a programmed “failure” rate^13,19^ such that the expected propagation through a population was slowed. These include inhibition by added domain fusions^20^, direct inhibition by anti-CRISPRs^21–24^, restriction on trafficking to and from the nucleus^13^, split nuclease systems^25^, and regulation of protein levels^13,26,27^.

Importantly, others have demonstrated that external cellular cues can be coupled to nuclease localization and/or activity^28–30^. For example, fusions to plasma-membrane localized G-protein coupled receptors (GPCRs) spatially restrict dCas9 until external ligand binding. When activated, a conformational change in the GPCR activates protease-dependent cleavage of the fused dCas protein, allowing it to be shuttled into the nucleus to modulate a downstream response^29^. At the organismal scale, external cues could include a range of stimuli including small molecules (pheromone), environmental changes (temperature, diet), or artificial means (small molecules). A recent study demonstrated that a domain-destabilized Cas9-based gene drive in flies could allow for a titration of drive activity; addition of an external cue stabilized the nuclease in a dose-dependent manner^27^. We envisioned a GD system where sensory information has been converted into an alteration to nuclease function (*e*_*W*_) and/or overall biological fitness (*f*).

In this study, we have expanded on a previously developed frequency-based population model^31^ to explore mechanisms to reverse traditional gene drives. We focus on use of inducible parameters for GD effectiveness, fitness, and a self-cleaving drive. We demonstrate that these designs could allow for a means of control that does not require the introduction of additional individuals. These findings could aid in development of an effective, safe, and fully reversible gene drive that could restore a native wild-type population.

## Results

### An inducible gene drive system (drive efficiency)

Previous gene drive models^31,32^ have examined the effects of drive efficiency (*e*_*W*_) and fitness (*f*). Our simulations of this same model^31^ illustrated the required initial parameters for both GD efficiency and fitness to allow for successful propagation (Fig. S2). We observed that a range of *e*_*W*_ values still resulted in GD takeover; however, there was a limit to the fitness cost that could be present within GD individuals despite values of *e*_*W*_ that approached one suggesting a much stronger effect of fitness on drive success. These data were also consistent with a previous individualistic GD model^32^ used to evaluate the contributions of *e*_*W*_ and *f*. In that study, the equation *f*(*e*_*W*_ + 1) > 1 represented scenarios where the GD was favored and would take over the population^32^.

**Figure 1:**
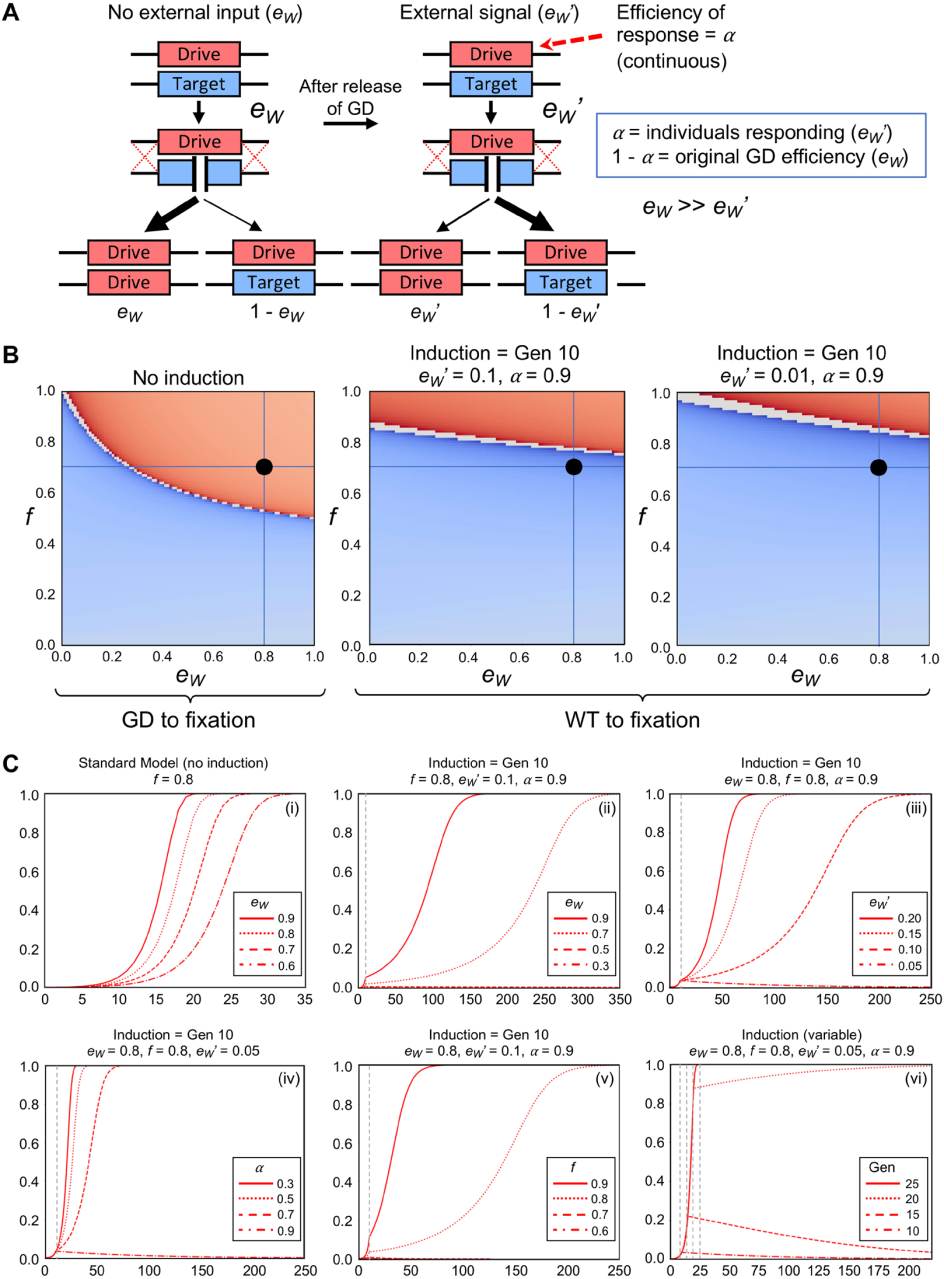
An inducible system to alter gene drive efficiency. (**A**) A theoretical GD system can translate an external signal into a change in GD effectiveness from initial *e*_*W*_ to a desired *e*_*W*_’. The portion of the population that successfully responded to the external cue was designated *α*; unaffected population was defined by (1 - *α*). The example illustrated assumed that the initial *e*_*W*_ was larger than the final *e*_*W*_’ following the shift (population reversion back to WT) and *α* was set to one. (**B**) A reduced *e*_*W*_’ allowed an initially successful GD to be overtaken by the WT population. Graphs plotting GD *e*_*W*_ and *f* (*left*, no induction) for an inducible system where *e*_*W*_’ was changed to 0.1 (*middle*) or to 0.01 (*right*) at generation 10 with an effectiveness of *α* = 0.9 are illustrated. A sample data point was highlighted with initial conditions of *e*_*W*_ = 0.8 and *f* = 0.7. The coloring scheme was identical to Fig. S2; GD to fixation (red) and WT to fixation (blue). Shading of colors illustrated the length of time required to reach fixation (darker, longer time). (**C**) Examination of varying initial parameters for a GD with inducible *e*_*W*_. Graphs (i-vi) illustrated GD allele frequencies in red. Population frequency was plotted on the y-axis and generation time was plotted on the x-axis. A single parameter was altered for each graph and the values tested were illustrated with line types (solid, dotted, dashed, and dash/dot). Graph (i) did not include an inducible mechanism. Vertical grey dotted lines indicated the starting point for application of the external signal.

Our model for an inducible gene drive expanded upon previous work^31^. We defined *e*_*W*_’ as the GD efficiency after introduction of the inducing agent and defined *α* as the success rate of the applied stimulus—the fraction of individuals within the population that successfully responded over a single generation (Fig. 1A). The first application of the signal occurred at generation 10 and continued for all subsequent generations. To illustrate one scenario for how this inducible GD could be applied, we modeled a system where the initial conditions (*e*_*W*_ and *f*) would allow the GD to move to fixation, but when exposed to the cue, the WT would be favored and the GD would be removed from the population (Fig. 1). Not all initial parameters allowed for such a shift—for example, conditions where *e*_*W*_ = 1 and *f* = 0.95 that were shifted to *e*_*W*_’ = 0.2, the GD was still favored to take over the population, albeit at a slower rate. However, for starting conditions of *e*_*W*_ = 0.8, *f* = 0.7, and *e*_*W*_’ = 0.1, the GD displayed a shift after application of the signal and this outcome was more potent when *e*_*W*_’ = 0.01 (Fig. 1B).

Using this model for inducible drive efficiency, we examined alteration of multiple variables within the simulation to determine which range of conditions allowed for a successful reversion to a wild population (Fig. 1C). In the standard model^31^, we illustrated the allele frequency of the gene drive (red) for varying conditions of *e*_*W*_ using a constant initial value for *f* (Graph i); all scenarios resulted in GD takeover. However, when the drive included an induced *e*_*W*_’ of 0.1 (Graph ii), only lower values of *e*_*W*_ allowed for the population to revert to wild type. These conclusions were based on a given set of initial parameters; these could shift depending on a number of input variables. One of the limitations of initial *e*_*W*_ values was due to the relatively high value of *e*_*W*_’ (0.1). Modeling the population trajectory for various *e*_*W*_’ values demonstrated that in order for the drive to revert to WT, this variable should have a very low value (Graph iii). Moreover, this was confirmed when we explored all combinations of *e*_*W*_ and *e*_*W*_’ using set values for *f* and *α* (Fig. S3). Another critical factor for this inducible system was the amount of the population impacted by the shift (*α*); this system required a high success rate for reversion (Graph iv). For all possible combinations of *α* and *e*_*W*_’ it was apparent that successful reversion would be achieved by simultaneously maximizing *α* and minimizing *e*_*W*_’ (Fig. S3). Drive fitness also had an effect; lower values of *f* allowed the system to remove GD individuals from the population following the inducible signal (Graph v). Finally, there remained some flexibility as to the timing of *e*_*W*_’ induction; application of the signal at generation 10 or 15 still allowed for full reversion to WT (Graph vi). Together, these data demonstrated that an inducible level of drive action could serve as a means to reverse a population to only WT individuals.

### An inducible gene drive system (individual fitness)

**Figure 2:**
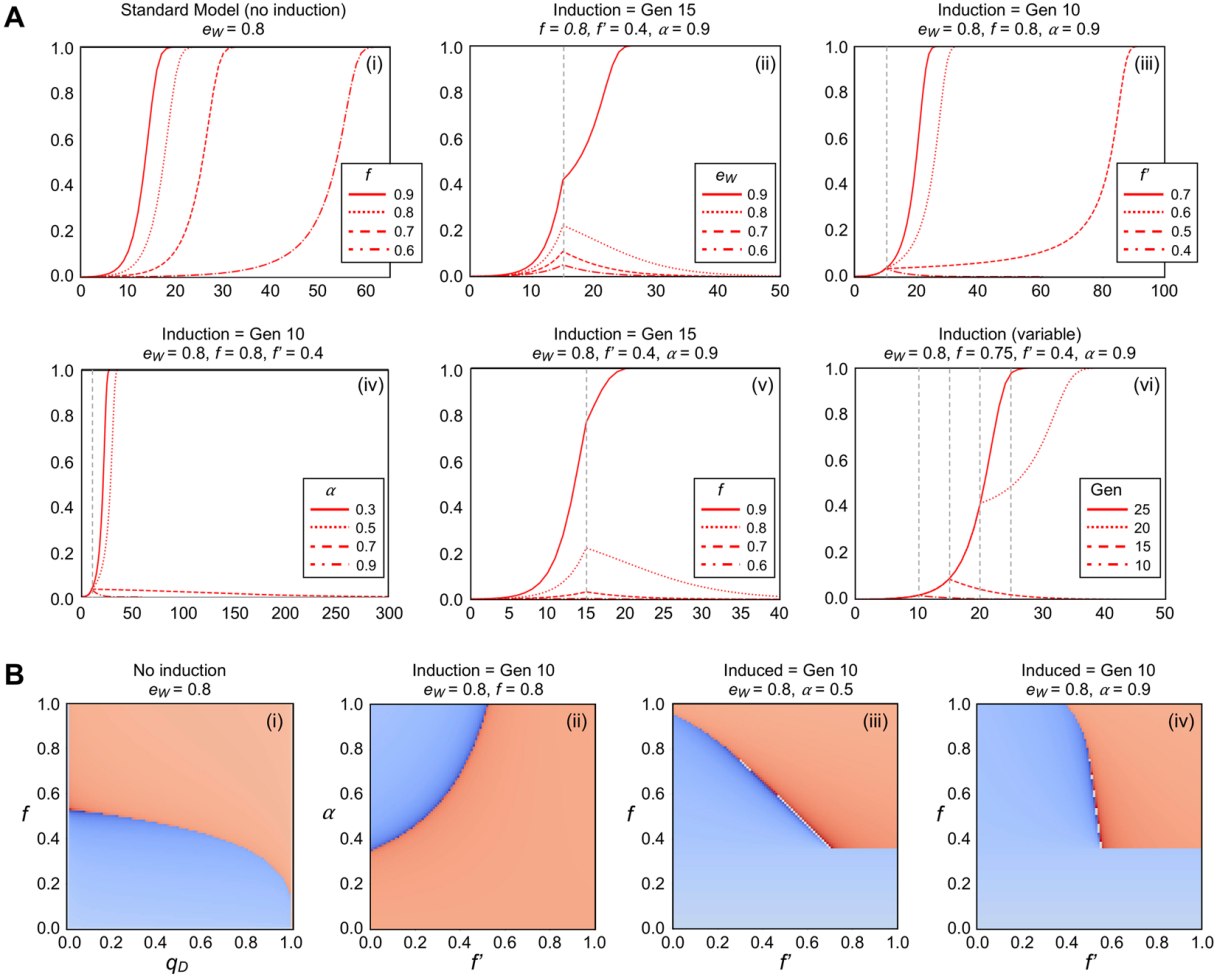
An inducible system to alter gene drive individual fitness. (**A**) Examination of both GD efficiency (*e*_*W*_) and fitness (*f* ) was explored for a drive system that included an inducible fitness parameter (*f* ’). The induction efficiency was denoted as *α*. Analysis of varying initial parameters for a GD with inducible *f*; graphs (i-vi) illustrated GD allele frequencies in red. Frequency was plotted on the y-axis and generation time was plotted on the x-axis. Line types corresponded to four tested values within each parameter. Graph (i) did not include an inducible mechanism. Vertical grey dotted lines highlighted the induction times used. (**B**) Four sets of simulations were performed (i-iv) comparing two parameters for GD systems with inducible *f*. Graph (i) did not include an inducible GD. The coloring scheme matched the simulations found in Fig. S2.

We also modeled an inducible system for altering individual GD fitness (Fig. 2). We envisioned that a reduction in fitness for GD individuals would be empirically determined and pre-programmed either within the drive locus or distant native loci (within split drives). We examined variation of initial conditions (Fig. 2A) similar to GDs with inducible drive efficiencies (Fig. 1C). In the standard model, a variety of initial *f* values allowed for successful GD takeover (Graph i). Population reversion was maintained for most *e*_*W*_ conditions with the exception of very high values. High *e*_*W*_ values prior to induction resulted in a critical gene drive frequency threshold being reached that, given the initial conditions, could not be reverted to WT (Graph ii). Altering the induced fitness parameter revealed that *f* ’ needed to be lowered to approximately 0.4 (for the given set of initial conditions) and this was in stark contrast to recommended levels for *e*_*W*_*’* (Graph iii). Along these lines, there was not a strict a requirement for values of *α*; 0.7 was sufficient to still revert the population to WT unlike for inducible *e*_*W*_ models (Graph iv). Moreover, a wider range of initial *f* values (0.8 and below) still allowed for this successful population shift (Graph v). Finally, the timing of the external signal needed to take place early within drive propagation (e.g. 10 or 15 generations); scenarios where the drive frequency was too high did not allow for a shift despite a high value of *α* (Graph vi).

We also examined the existing (or initial) frequency of drive alleles within a population and drive fitness independent of any inducible system (Fig. 2B, Graph i). For our simulations, we included an initial GD frequency of 0.001; this translated into a requirement of GD fitness of greater than approximately 0.5. However, as the frequency of GD alleles increased within the population, the drive fitness could be lowered, and this still resulted in an overall GD takeover. Also, the model with inducible *f* allowed for a broader range of values for both *α* (as low as 0.4) and *f’* (nearly 0.5) compared with *e*_*W*_’ that would still allow for a population reversion to WT (Fig. 2B, Graph ii). Finally, comparison of *f* and *f’* at two values for *α* (Graphs iii and iv) demonstrated that when a larger proportion of individuals responded to the external signal (*α* = 0.9), the initial value of *f* became less significant (Fig. 2B). For a specific set of initial conditions (*e*_*W*_ = 0.8, *f* < 0.3), the simulation assigned the WT because the GD was removed from the population prior to induction at generation 10. Interestingly, our comparisons also highlighted an *increase* in GD fitness for certain combinations of *f* and *f’* such that an inducible *f* gain could allow a GD to propagate despite a low initial fitness <0.5. Together, these data illustrate the potency of alteration of drive fitness across a range of initial conditions compared to inducible drive efficiency.

### Modeling an inducible self-cleaving gene drive

**Figure 3:**
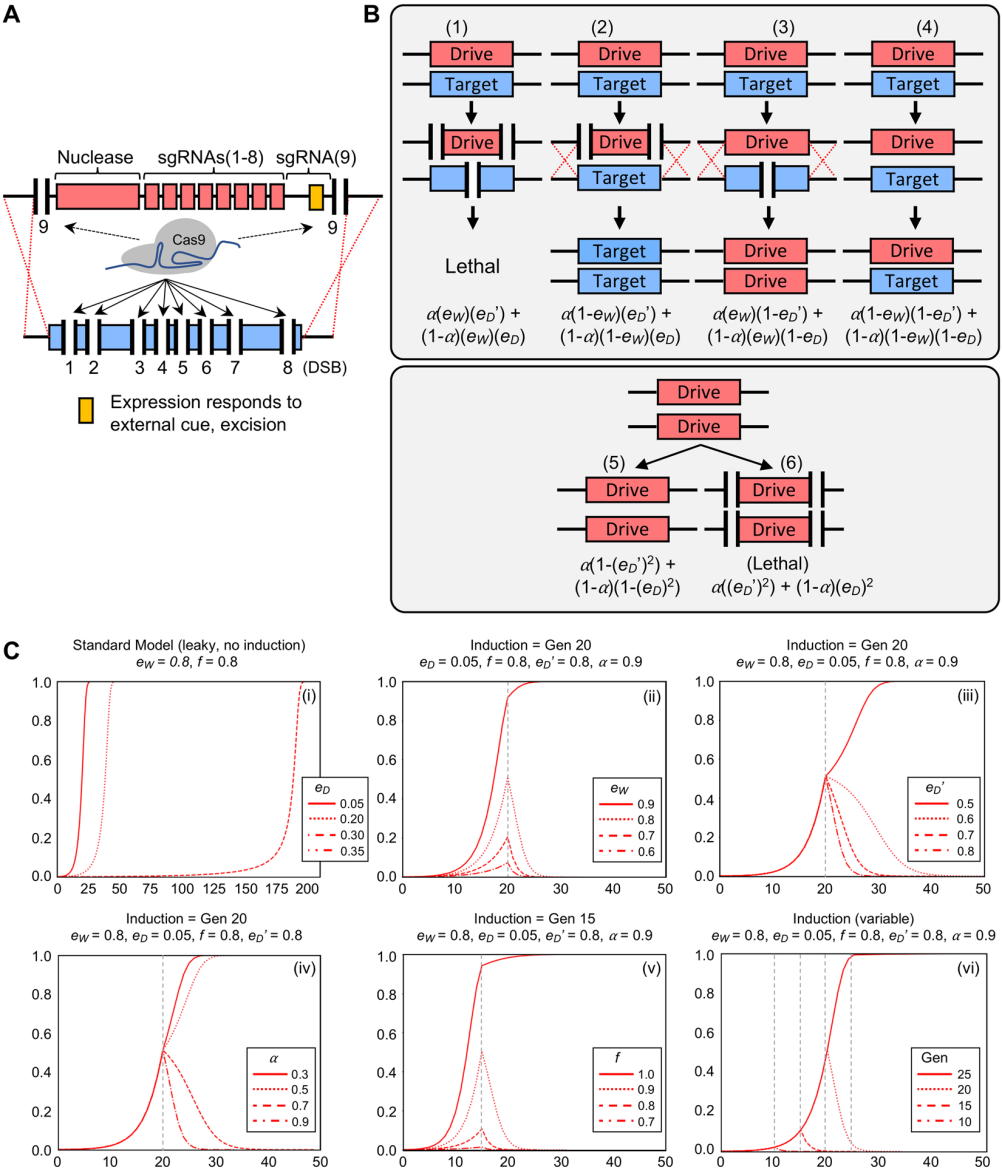
An inducible self-cleaving gene drive system. (**A**) Schematic of a GD arrangement that includes an inducible guide RNA cassette—sgRNA(9)—that would activate self-cleavage of the drive itself. (**B**) Two GD efficiencies were modeled: (i) *e*_*W*_ represented the ability to cleave the intended WT target and (ii) *e*_*D*_ represented the ability to self-target the drive cassette. Four scenarios were outlined (1–4) between pairing of the GD and WT alleles that resulted in a lethal phenotype, homozygous WT, homozygous GD, and heterozygous GD/WT genotypes. Activation of *e*_*D*_ prior to activation of the external cue (e.g. “leaky” activation) was included within the model; *e*_*D*_*’* represented self-cleavage after the induction event. The outcomes of a homozygous GD/GD pairing were also modeled (scenarios 5–6). (**C**) Graphs (i-vi) illustrated drive allele frequencies in red. The allele frequency was plotted on the y-axis and the generation time was plotted on the x-axis. Line types (solid, dotted, dashed, and dash/dot) corresponded to four values sampled for each simulation. Graph (i) did not include an inducible element; a basal “leaky” value of *e*_*D*_ was set to various values at generation 0. Vertical grey dotted lines specified application of the external signal.

While an inducible GD system to modulate *e*_*W*_ or *f* provides a suite of options, we reasoned that a different regulatory system could be employed to more rapidly remove GD individuals from a population. Therefore, we designed a theoretical GD that would include a self-cleaving module (Fig. 3A). One possible architecture could include an inducible guide RNA cassette that would target the nuclease to sites flanking the drive itself. Alternatively, multiple nucleases might be employed that utilize distinct and non-compatible guide RNAs with the same effect. In our model, we have included only a single nuclease type such that GD efficiency was identical for all GD actions. Here, *e*_*W*_ represented the efficiency for cleaving the WT target (and copying of the drive via HDR) and *e*_*D*_ represented the efficiency for self-cleaving the GD locus (and subsequent repair) (Fig. 3B). For pairings between the drive and WT, we modeled four separate outcomes: (i) dual cleavage of both the target and drive, (ii) cleavage of only the drive, (iii) cleavage of only the target, and (iv) no cleavage resulting in a heterozygous individual. We reasoned that in our GD design, dual cleavage events to both chromosomes (and removal of any programmed essential gene) would be lethal and that single cleavage of only the GD would present a unique scenario where the WT gene would be doubled to recreate a homozygous WT individual. For existing GD individuals in the population, successful activation of the self-cleavage module could result in excision of both alleles and a lethal phenotype.

In our model, we assumed that both self-cleaving events occurred at rates *e*_*D*_ or *e*_*D*_’ (after induction) and that HDR-based repair was responsible for repair following DNA cleavage. Finally, we also included calculation of a “leaky” system where an initial value of *e*_*D*_ existed (typically 0.05) prior to activation of the external signal *α*. This was to test whether a small amount of self-cleavage would affect the initial propagation of the GD system and represented the reasonable biological assumption that even tightly regulated promoters might allow for a small level of transcript (in the case of either the regulated self-targeting guide RNA and/or second nuclease). For a given set of initial conditions, we modeled increasing levels of *e*_*D*_ in a system that did not include any inducible signal (Fig. 3C, Graph i). Interestingly, the system was robust for “leaky” levels of self-cleavage up to 0.3; the overall effect was the shift in the length of time required for GD takeover.

Modeling of the inducible self-cleaving system demonstrated a variety of initial parameters could be accommodated that still resulted in successful population reversion to WT (Fig. 3C). Values of *e*_*W*_ up to 0.8 were still tolerated (Graph ii) as well as induced *e*_*D*_’ values as low as 0.6 (Graph iii). Similar to the application success rate for inducible *f*, values for *α* did not need to be extremely high (Graph iv). Furthermore, drives with fitness values up to 0.9 could still be reverted using this system (Graph v). We observed that activation of this system could be relatively late (generation 20) compared with the length of time required for GD takeover for a given simulation and the allele frequency decline was much more rapid than compared with models for inducible *e*_*W*_ or *f* (Graph vi). We also expanded our analysis of values for *e*_*W*_, *e*_*D*_’, and induction time which would account for varying values between *e*_*W*_ and *e*_*D*_*/e*_*D*_’ (Fig. S4). We found that this type of self-cleaving system could still allow for reversion to WT even when the induced *e*_*D*_’ was identical to *e*_*W*_ (for single-nuclease drives) or for scenarios where *e*_*D*_’ < *e*_*W*_ (for dual nuclease drives with differences in efficiency). Together, these data demonstrated an alternative mechanism for inducible GD population suppression that provides a unique set of requirements for GD strength and fitness.

## Discussion

Few studies have proposed mechanisms by which removal of GD organisms could be accomplished once already released. Current ideas include gene drives that counter other gene drives (anti-drives)^33,34^, drives with built-in limitations (daisy-chain drives)^35,36^, or underdominance drives (UD)^35,37–39^. In the case of the anti-drives, a standard reversal system only targets GD individuals; immunizing anti-drives are able to target both GD and WT individuals. For some scenarios, AD systems may not eliminate drives within a population, and, instead, might achieve a stable equilibrium^33^. Moreover, without additional modifications, AD systems require construction at the same locus that the GD was originally installed. This might prove challenging in some scenarios where anti-drives are not already engineered and available for release. Daisy-chain systems can provide local spread of a drive element but they cannot propagate at the same scale as traditional drives; the goal is limited spread of drives, rather than targeted removal of active drives^36^. Finally, in the case of UD systems, proposals for population reversal requires the introduction of either wild-type individuals^40^ or “free suppressor” individuals^38^. Therefore, we focused on novel drive systems that might provide a variable level of population control and ultimately allow for reversion to the original WT species without the need for a secondary release of modified (or wild-type) individuals.

Previous theoretical models have included a metric for gene drive efficiency (*e*, represented in our work as *e*_*W*_). In our system, we assumed that the two requirements of GD action—cleaving the target(s) and subsequent DNA repair via HDR—were coupled together. Programmed values of *e*_*W*_ less than 1 would result in a predictable frequency of heterozygous individuals without any resistant allele(s). Titration of *e*_*W*_ has the advantage of being generally applicable to other eukaryotic systems. For instance, nucleocytoplasmic transit (of Cas9) is widely conserved across species and expression of the anti-CRISPR peptides would be predicted to function regardless of the cell type or organism. In its simplest form, application of a gene drive where *e*_*W*_ < 1 would provide additional time to implement changes or release and distribute countering agents. However, as our model illustrates, use of an inducible *e*_*W*_ to favor return of the WT population after GD release would require an external signal to be recognized by the entire population. While this is certainly feasible when using naturally occurring events (temperature, environmental conditions, etc.), this would present additional challenges for delivery and application, but could certainly be attempted on isolated small-scale field trials or laboratory settings.

We also recognize that the identity of the external signal would have its own challenges and/or potential costs. Ideally, delivery of the signal would have a minimal or negligible effect on the surrounding ecosystem (and human health in the case of agricultural application). We found a much stronger effect of altering the overall fitness of the organism. However, the precise genetic target and mechanism for altering *f* would likely be species-specific and may be difficult to quantify. However, titration of *f* allowed for a lower overall sample of the population to respond to an added cue compared to *e*_*W*_. While others have demonstrated that installation of drives sometimes includes an overall fitness cost, our data demonstrate that there are levels of *f* that can still allow for GDs to effectively propagate through a population similar to previous findings^41^. Along these lines, previous studies have recommended that “responsible” gene drive systems have a purposefully reduced fitness^42^.

Our proposal for an inducible self-cleaving gene drive system would provide multiple benefits in terms of regulation and drive reversal. For one, cleavage of the GD without cleavage of the WT target allele would provide a unique scenario where the WT locus was copied to replace the gene drive. This provides a more potent option than either a reduction of drive efficiency, drive fitness, or even use of an anti-drive because it (i) removes the GD allele directly and (ii) simultaneously increases the proportion of WT alleles within the population (without any supplementation of additional individuals). While our system demonstrated that use of an additional guide RNA cassette could be activated using the same nuclease, two separate CRISPR systems could be employed such that the level of effectiveness for *e*_*W*_ and *e*_*D*_/*e*_*D*_’ could be tuned accordingly. Moreover, our findings illustrated that this type of drive system required a lower rate of response to an external cue compared to a switch in *e*_*W*_’ and could still serve as an effective GD despite a high basal level of *e*_*D*_ activation and self-removal of a fraction of the population. Coupling separate inducible systems together (such as self-cleavage, GD fitness, and/or *e*_*W*_) could provide multiple independent mechanisms to control drive propagation and allow for targeted drive removal should the need arise.

While a complex gene drive that allows for external input may face technical challenges in design and construction, the goal of this study was to explore theoretical systems that would specifically address the issues of containment and reversal/removal (Table 1). We envision that these types of safety mechanisms would provide additional levels of control and programmability that are not currently possible in simple GD setups that are designed with only initial parameters and a single outcome. Furthermore, failsafe systems to protect the original WT species, even if never used in application, are a critical step towards gaining support for future laboratory research and possible application of gene drives within native ecosystems.

**Table 1:** Summary of proposed gene drive designs and potential benefits and challenges.

Gene Drive Strategy	Description and Outcomes	Pros/Cons^a^
1. Reduced initial *e*_*W*_ and/or *f*	Slowed drive propagation; sensitized GD population	Requires identification of conserved mechanisms for titration of *e*_*W*_. Species-specific means to reduce (and quantify) fitness. Cannot alter population once released. Pre-determined outcome for gene drive success.
2. Inducible *e*_*W*_	Ability to prevent GD take over, revert population to WT	Requires significant induction success rate (*α*) and very low value for *e*_*W*_*’*. Requires continuous application.
3. Inducible *f*	Ability to prevent GD take over, revert population to WT	Requires lower success rate and less severe reduction in *f’*. Likely species-specific mechanism. Requires continuous application.
4. Inducible self-cleaving GD	Direct removal of GD alleles without the need for an anti-drive system, reversion to WT population	Allows for differences in *e*_*W*_ and *e*_*D*_/*e*_*D*_*’* for degree of GD removal. Does not require a high induction rate *α*. System is robust against modest degree of inappropriate activation and self-cleavage. Requires continuous application.

^a^The advantages and challenges for each system are discussed. These are not focused on challenges of design and creation of the intended drive, but rather on drive application and potential uses.

## Methods

### Population models for inducible gene drive efficiency and fitness

We expanded on a previous frequency-based model (illustrated below) of gene drive population dynamics; this model assumed random mating, non-overlapping generations and an infinite population size^31^. Our modifications to this equation introduced new variables for our inducible and self-cleaving designs (*e*_*W*_’, *f* ’, *e*_*D*_, and *e*_*D*_’).

$${q}_{D}^{^{\prime} }=\frac{f({q}_{D}^{2}+2{q}_{W}{q}_{D}{e}_{W})+(1-h+f\,h)({q}_{W}{q}_{D}(1-{e}_{W}))}{1-(1-f)({q}_{D}^{2}+2{q}_{W}{q}_{D}{e}_{W})-(h-f\,h)2{q}_{W}{q}_{D}(1-{e}_{W})}$$

#### Frequency-based model

We assumed no GD-resistance of any kind (including no NHEJ-based repair and subsequent resistance). For the standard model, we included two allele types, *W* and *D*, which denoted wild-type and gene drive with frequencies *q*_*W*_ and *q*_*D*_, respectively. The summation of these frequencies always totaled to one. This resulted in three distinct classes; homozygous wild-type, homozygous gene drive, and the heterozygote between wild-type and gene drive. The success rate of the GD, the ability to both cleave WT target DNA and copy the drive cassette to the homologous chromosome, was denoted by *e*_*W*_. While we recognize that alternate scenarios exist (such as DNA cleavage without copying of the drive), these would be accounted for in the overall design of the drive itself—NHEJ-based repair in the absence of copying of the drive would result in individuals that would be sterile or inviable (see Fig. S1). Failure of the drive was denoted by (1 − *e*_*W*_).

#### Heterozygote fitness

The gene drive homozygote had an associated fitness relative to wild-type that was represented by the variable *f*. The fitness of the heterozygote was calculated using a degree of dominance, *h*; this was represented by (1 − *h)* + *fh*. For all simulations, the fitness of wild type individuals was set to one and *h* was set to 0.5. For heterozygous individuals (WT/GD) with an active drive system, the genotype was immediately converted to the homozygous state (GD/GD) and the corresponding fitness of the individual was calculated based on this genotype.

#### Applied external signal

Our equations for inducible drives also introduced the variable *α* which represented the fraction of individuals affected by the external inducing agent. This value was shifted from zero to a specific level at a predetermined generation (the shift occurred within one generation). The inducing agent was then continuously applied at the same level for every subsequent generation after its initial release.

#### Simulation parameters

The initial frequency of the GD was set to 0.1% of the total population. Simulations were run until the population achieved (i) fixation of the GD, (ii) loss of the GD, or (iii) the maximum number of generations (1,000) was reached. We declared the population had reached fixation when any allele had a frequency >0.99999. Graphics utilized a 2D graphics package^43^. The allele frequencies of the next generation (*q*_*W*_’ and *q*_*D*_’) were calculated by the following equations, where *q*_*W*_’ = 1 − *q*_*D*_’.

Equation for inducible efficiency

1 $${q}_{D}^{^{\prime} }=\frac{f({q}_{D}^{2}+2{q}_{W}{q}_{D}(\alpha {e}_{W}^{^{\prime} }+(1-\alpha ){e}_{W}))+(1-h+f\,h)({q}_{W}{q}_{D}(1-(\alpha {e}_{W}^{^{\prime} }+(1-\alpha ){e}_{W})))}{1-(1-f)({q}_{D}^{2}+2{q}_{W}{q}_{D}(\alpha {e}_{W}^{^{\prime} }+(1-\alpha ){e}_{W}))-(h-f\,h)2{q}_{W}{q}_{D}(1-(\alpha {e}_{W}^{^{\prime} }+(1-\alpha ){e}_{W}))}$$

Equation for inducible fitness

2 $${q}_{D}^{^{\prime} }=\frac{(\alpha f^{\prime} +(1-\alpha )f)({q}_{D}^{2}+2{q}_{W}{q}_{D}{e}_{W})+(1-h+(\alpha f^{\prime} +(1-\alpha )f)h)({q}_{W}{q}_{D}(1-{e}_{W}))}{1-(1-(\alpha f^{\prime} +(1-\alpha )f))({q}_{D}^{2}+2{q}_{W}{q}_{D}{e}_{W})-(h-(\alpha f^{\prime} +(1-\alpha )f)h)2{q}_{W}{q}_{D}(1-{e}_{W})}$$

Model for self-cleaving gene drive system

The model for the inducible self-excising drive introduced new variables *e*_*D*_ and *e*_*D*_’. The presence of *e*_*D*_ (initial self-cleavage of the gene drive) existed to allow for any possible activation prior to exposure to the inducing agent (e.g. “leaky” inducible system) while *e*_*D*_’ represented the intentional self-cleaving activation.

3 $${q}_{D}^{^{\prime} }=\tfrac{f({q}_{D}^{2}(1-{((1-\alpha ){e}_{D}+\alpha {e}_{D}^{^{\prime} })}^{2})+2{q}_{W}{q}_{D}((1-\alpha ){e}_{D}+\alpha {e}_{D}^{^{\prime} })(1-{e}_{W}))+(1-h+f\,h){q}_{W}{q}_{D}(1-((1-\alpha ){e}_{D}+\alpha {e}_{D}^{^{\prime} }))(1-{e}_{W})}{\begin{array}{c}1-(1-f)({q}_{D}^{2}(1-{((1-\alpha ){e}_{D}+\alpha {e}_{D}^{\text{'}})}^{2})+2{q}_{W}{q}_{D}(1-((1-\alpha ){e}_{D}+\alpha {e}_{D}^{^{\prime} })){e}_{W})\\ -(h-f\,h)2{q}_{W}{q}_{D}(1-((1-\alpha ){e}_{D}+\alpha {e}_{D}^{^{\prime} }))(1-{e}_{W})-\,{q}_{D}^{2}{((1-\alpha ){e}_{D}+\alpha {e}_{D}^{^{\prime} })}^{2}-\,2{q}_{W}{q}_{D}{e}_{W}((1-\alpha ){e}_{D}+\alpha {e}_{D}^{^{\prime} })\end{array}}$$

### Animal and human subject statement

This study does not use any animals or human subjects.

## Supplementary information

Supplementary Information

## Data Availability

The datasets generated during and analyzed during the current study are available from the corresponding author on reasonable request for research purposes.
