# Supplementary material for: Mathematical modeling of self-contained CRISPR gene drive reversal systems
Source: Sci Rep. 2019 Dec 27;9:20050. doi: 10.1038/s41598-019-54805-8 (PMC6934693; doi:10.1038/s41598-019-54805-8)
Supplement: Supplementary file 1 — Supplementary Information [file 41598_2019_54805_MOESM1_ESM.docx]

**SUPPORTING INFORMATION**

For

**Mathematical modeling of self-contained CRISPR gene drive reversal systems**

Matthew G. Heffel^1^ and Gregory C. Finnigan^2*^

^1^Division of Biology, 116 Ackert Hall, Kansas State University, Manhattan, KS 66506 USA

^2^Department of Biochemistry and Molecular Biophysics, 141 Chalmers Hall, Kansas State University, Manhattan, KS 66506 USA

*Correspondence to: Gregory C. Finnigan, Phone: (785) 532-6939; FAX; (785) 532-7278;

E-mail: [gfinnigan@ksu.edu](mailto:gfinnigan@ksu.edu)

Running title: Mathematical modeling of CRISPR gene drives

Keywords: CRISPR, Cas9, gene drive, biotechnology, computational modeling, reversal systems

**
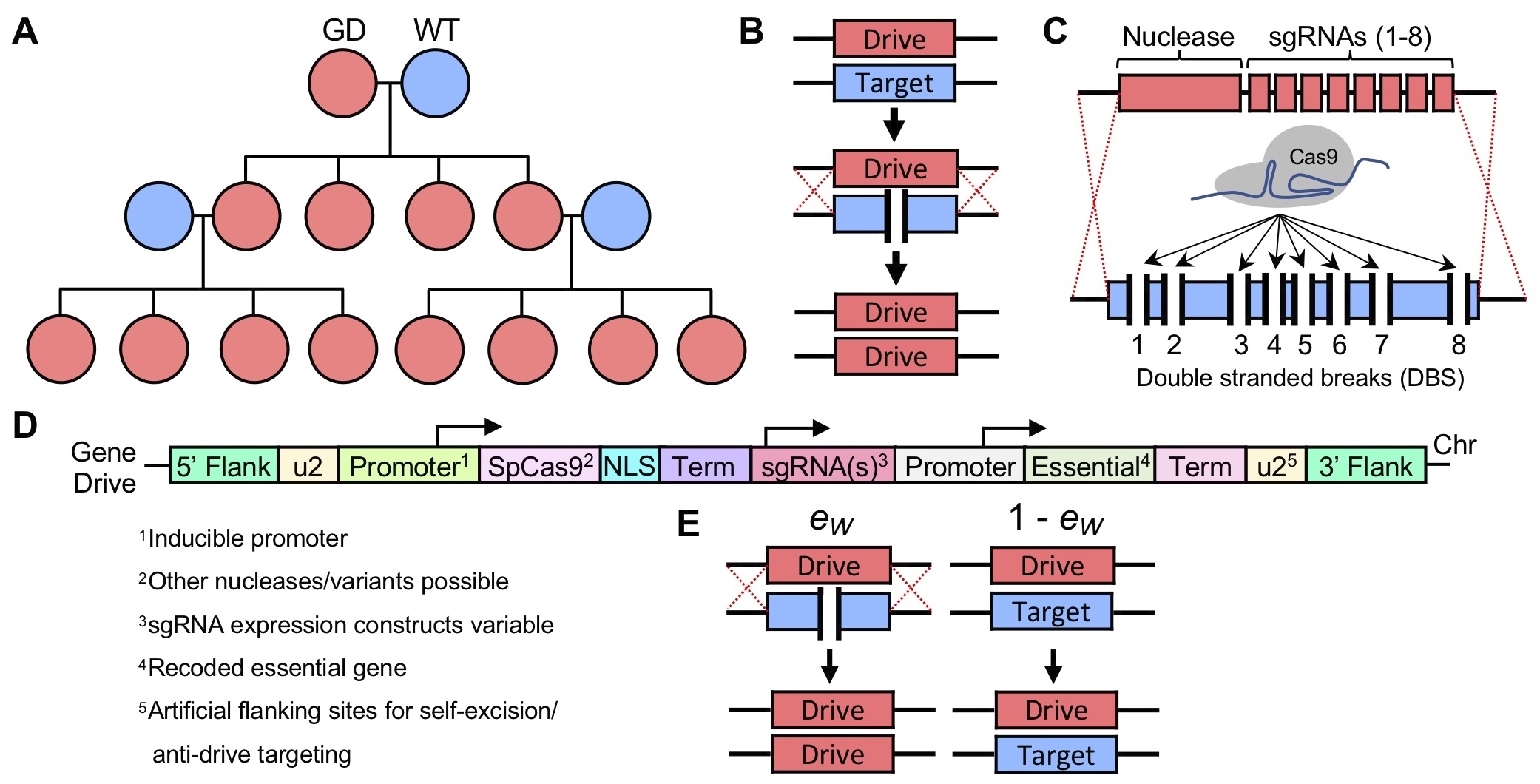
**

**Supplemental Figure S1.** Design of a tunable CRISPR gene drive system. (A) *Super*-Mendelian inheritance of an artificial homing drive. GD individuals (red), when paired with WT (blue), result in 100% of progeny in the homozygous state. (B) Mechanism of drive propagation involves the drive construct creating a DSB within the target (WT) allele; repair via HDR of the DNA break using the gene drive construct as the source of donor DNA material. (C) A proposed GD harboring multiple guide RNA constructs. The GD consists of at least one nuclease gene (e.g. *S. pyogenes* Cas9) and multiple sgRNAs with the intended targets present within the corresponding WT locus. (D) An example GD construct might also include a re-coded essential gene (immune to action of the drive) along with flanking artificial sites (u2)^1^ that could be self-targeted (see Fig. 3) or cleaved by a separate reversal drive/system at a later date. (E) Efficiency of the GD (*e_W_*) represented the ability of the drive to both cleave the target (blue) and copy the entirety of the drive (red). The quantity (1 - *e_W_*) represented a scenario where there was no DNA cleavage resulting in a heterozygous individual (GD/WT) with no alteration of the target (and no generation of resistant alleles).

**
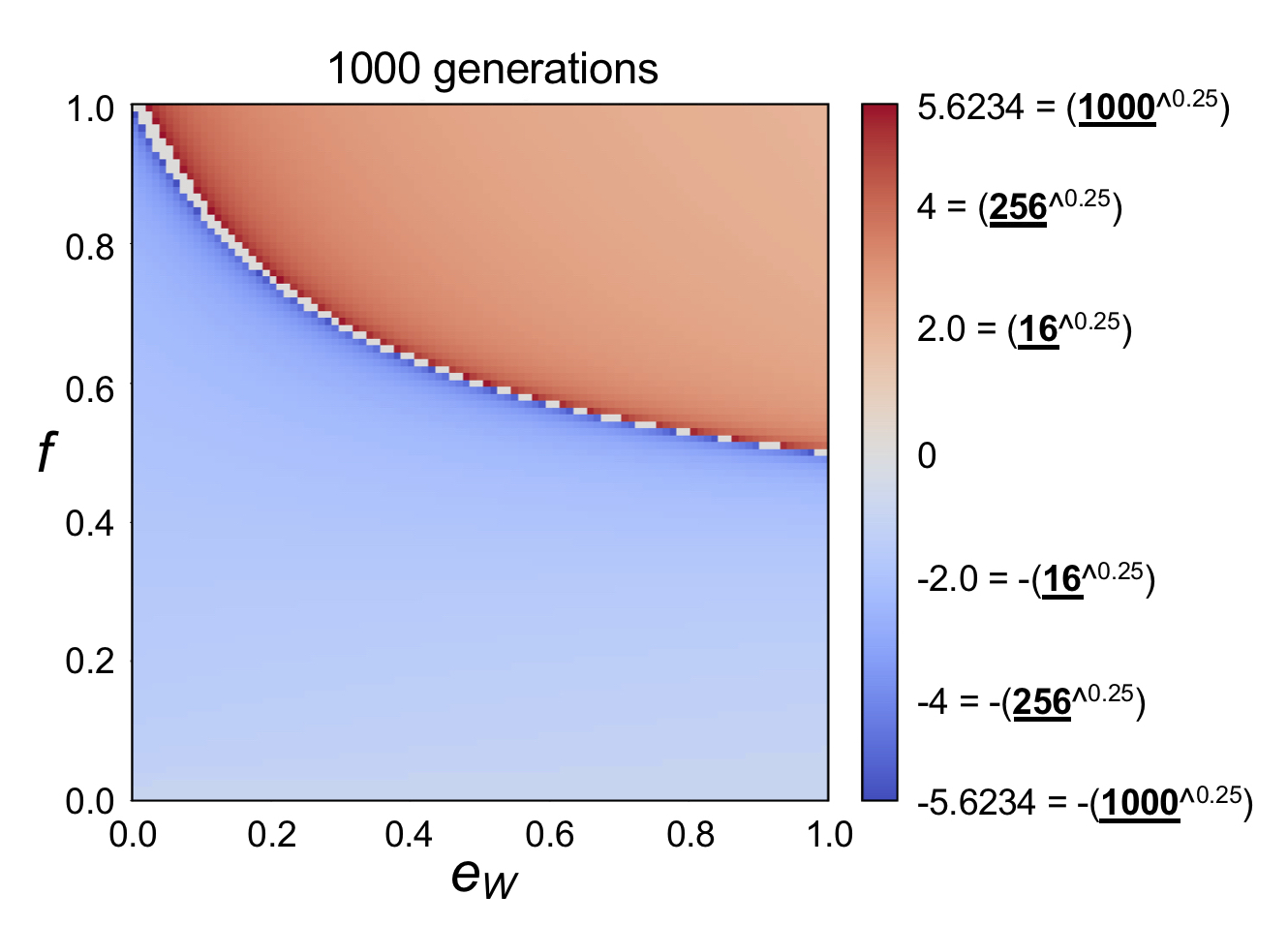
**

**Supplemental Figure S2.** Modification of CRISPR gene drive efficiency and fitness in a previous^2^ frequency-based model for population dynamics. Simulations utilized a previous mathematical model with very similar results^2^. The fitness of heterozygotes was defined as the average fitness of WT and GD individuals (degree of dominance = 0.5). Modeling of values of initial parameters of *e_W_* and *f* demonstrated whether the GD (red) or WT (blue) population moved to fixation by the end of the simulation. If the simulation (1000 generations) had not completed (one allele frequency reached >0.99999), boxes were colored grey. The shade of color illustrated the length of time (generations) required for each population to reach fixation (darker, longer time; lighter, shorter time). Values illustrated are the generation time (bold, underlined) raised to the ¼ power.

**
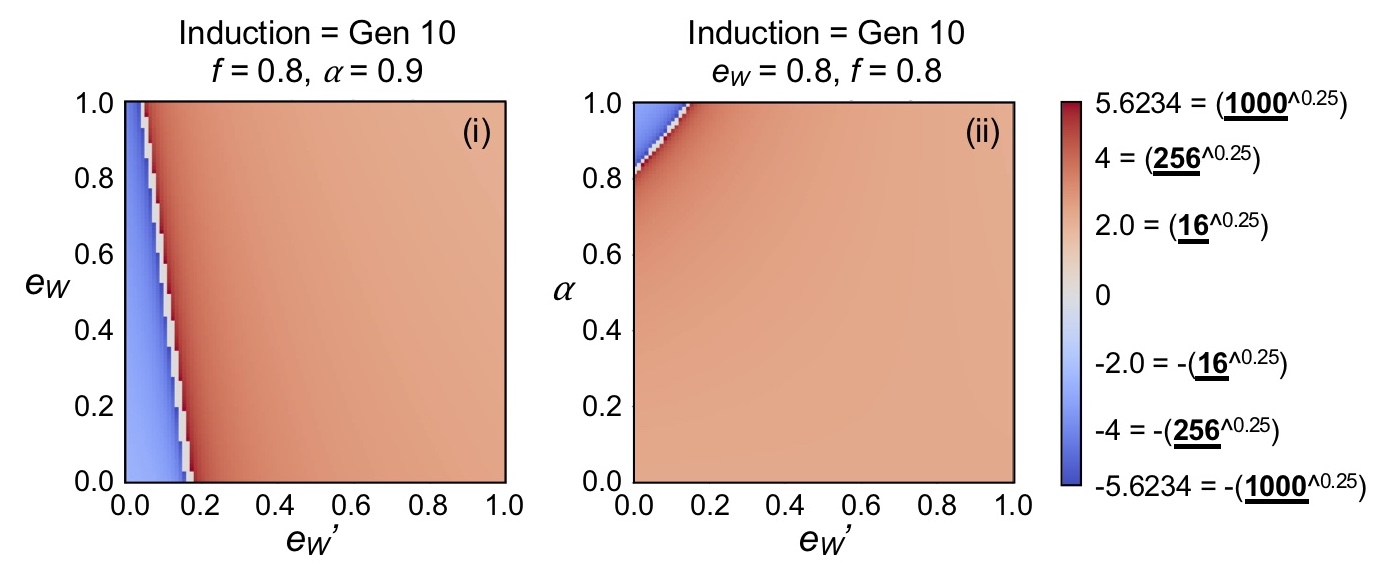
**

**Supplemental Figure S3.** Parameter considerations for gene drives with an inducible *e_W_.* Graph (i), combinations between initial *e_W_* and induced *e_W_’* values (generation 10) were modeled for gene drives with *f* = 0.8 with an activation rate *α* = 0.9. The coloring scheme was identical to Fig. S2. From these initial conditions, the recommendation would be to either (i) minimize the value of *e_W_’* following induction and/or (ii) reduce the strength of *e_W_*. However, since changing the latter results in a much slower drive (increasing generations) to population takeover, decreasing the induced value of *e_W_’* would likely provide the most effective option that could accommodate a variety of initial conditions. Graph (ii), both activation rate (*α*) and *e_W_’* were examined for a GD with initial parameters of *e_W_* = 0.8 and *f* = 0.8. These data illustrated the importance of activation of a large number of the GD individuals (> 0.8). However, decreasing the induced value of *e_W_’* could allow a small amount of flexibility. Together, these simulations highlight that gene drives with an inducible *e_W_* should include a high value for *α* and a correspondingly low value for *e_W_’* to maximize the success for population reversion back to WT.

**
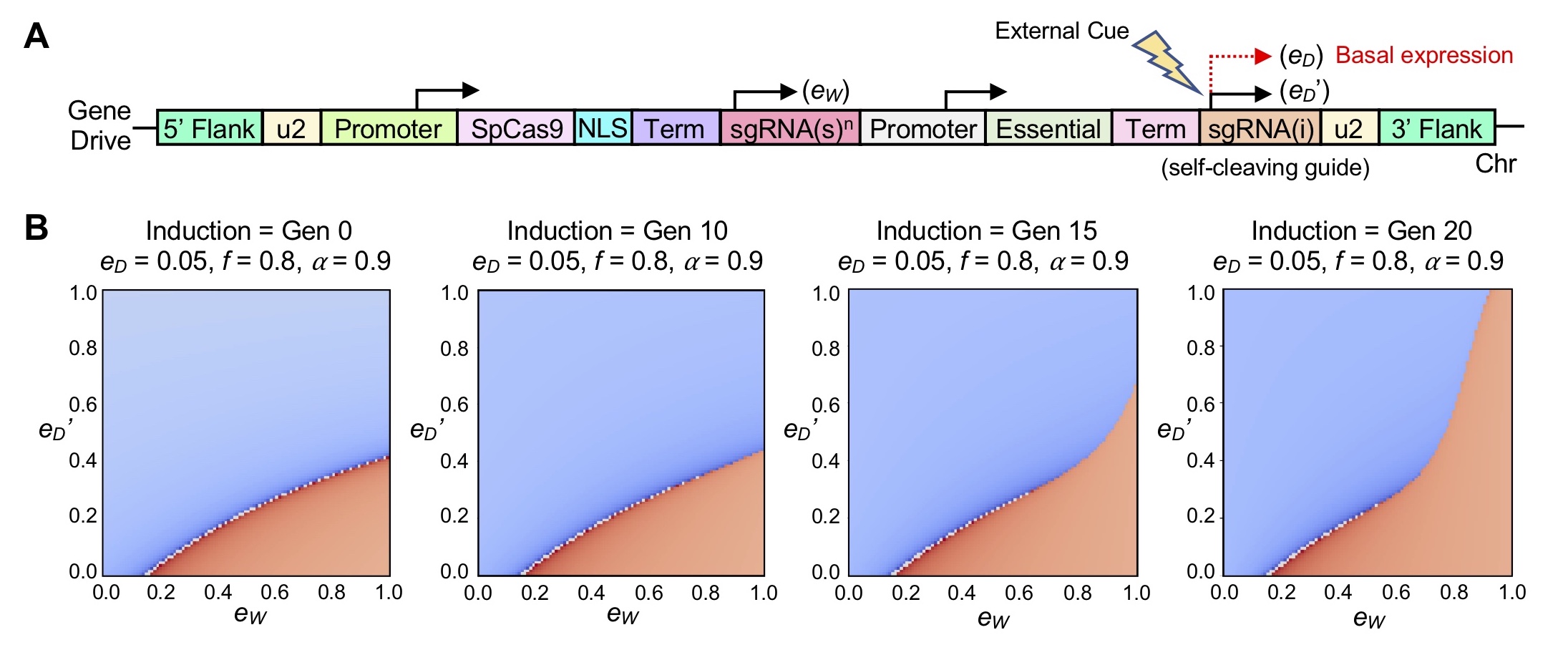
**

**Supplemental Figure S4**. Basal levels of self-cleavage of an inducible gene drive system still allowed for potent GD action. (A) Proposed schematic for a GD system including “standard” sgRNA(s) for action of the drive (*e_W_*) as well as a separate inducible sgRNA(i) responsible for self-cleavage of the drive at unique flanking target sites (u2).^1^ Basal activity resulting from the self-cleaving guide was designated as *e_D_* whereas activation of the construct (after application of the external signal) was denoted *e_D_’*. We recognize that an alternative design could include dual nucleases—one to propagate the drive (*e_W_*) and a second to only be used for self-cleavage (*e_D_*/*e_D_’*). As illustrated in this example using only *S. pyogenes* Cas9, the values for both *e_W_* and *e_D_’* would likely be similar or equivalent, but additional CRISPR nucleases or other modifications would allow distinct values for recruitment and/or cleaving of the target or drive cassette itself. (B) Simulations were performed examining the strength of the gene drive (*e_W_*) and strength of the induced self-cleaving guide (*e_D_’*). Initial parameters for these models included a standard drive where *f* = 0.8, *α* = 0.9, and a consistent level of “leakiness” was set at *e_D_* = 0.05. The coloring scheme used to designate gene drive (red) or WT (blue) takeover was identical to those from Fig. S2. The simulations were performed using activation times at generations 0, 10, 15, and 20. Delaying the timing of activation of the self-cleaving *e_D_’* caused a shift in the number of conditions that maintained GD takeover (red), including for high values of *e_D_’*. These data demonstrated that a self-cleaving system is (i) robust against changes in activation time, (ii) can allow for successful reversion to WT even when *e_D_’* < *e_W_*, (iii) can operate across a range of *e_W_* values, and (iv) could still function when *e_D_’* = *e_W_* (for single nuclease systems).

**REFERENCES**

1 Finnigan, G. C. & Thorner, J. mCAL: a new approach for versatile multiplex action of Cas9 using one sgRNA and loci flanked by a programmed target sequence. *G3 (Bethesda, Md.)* **6**, 2147-2156, doi:10.1534/g3.116.029801 (2016).

2 Deredec, A., Burt, A. & Godfray, H. C. The population genetics of using homing endonuclease genes in vector and pest management. *Genetics* **179**, 2013-2026, doi:10.1534/genetics.108.089037 (2008).
